# Deleterious mutations predicted in the sorghum (*Sorghum bicolor*) *Maturity* (*Ma*) and *Dwarf* (*Dw*) genes from whole-genome resequencing

**DOI:** 10.1038/s41598-023-42306-8

**Published:** 2023-10-03

**Authors:** Nathan P. Grant, John J. Toy, Deanna L. Funnell-Harris, Scott E. Sattler

**Affiliations:** 1 Wheat, Sorghum and Forage Research Unit, Agricultural Research Service, United States Department of Agriculture, Lincoln, NE USA; 2 Department of Agronomy and Horticulture, University of Nebraska-Lincoln, Lincoln, NE USA; 3 Department of Plant Pathology, University of Nebraska-Lincoln, Lincoln, NE USA

**Keywords:** Plant breeding, Genetic variation, Plant breeding

## Abstract

In sorghum [*Sorghum bicolor* (L.) Moench] the *Maturity* (*Ma1*, *Ma2*, *Ma3*, *Ma4*, *Ma5*, *Ma6*) and *Dwarf* (*Dw1*, *Dw2*, *Dw3*, *Dw4*) loci, encode genes controlling flowering time and plant height, respectively, which are critical for designing sorghum ideotypes for a maturity timeframe and a harvest method. Publicly available whole-genome resequencing data from 860 sorghum accessions was analyzed in silico to identify genomic variants at 8 of these loci (*Ma1*, *Ma2*, *Ma3*, *Ma5*, *Ma6*, *Dw1*, *Dw2*, *Dw3*) to identify novel loss of function alleles and previously characterized ones in sorghum germplasm. From ~ 33 million SNPs and ~ 4.4 million InDels, 1445 gene variants were identified within these 8 genes then evaluated for predicted effect on the corresponding encoded proteins, which included newly identified mutations (4 nonsense, 15 frameshift, 28 missense). Likewise, most accessions analyzed contained predicted loss of function alleles (425 *ma1*, 22 *ma2*, 40 *ma3*, 74 *ma5*, 414 *ma6*, 289 *dw1*, 268 *dw2* and 45 *dw3*) at multiple loci, but 146 and 463 accessions had no predicted *ma* or *dw* mutant alleles, respectively. The *ma* and *dw* alleles within these sorghum accessions represent a valuable source for manipulating flowering time and plant height to develop the full range of sorghum types: grain, sweet and forage/biomass.

## Introduction

Sorghum (2n = 20; ~ 730 Mb) is the fifth most significant cereal grain crop in production behind maize, rice, wheat and barley (USDA-NASS; www.nass.usda.gov). Originating from tropical and subtropical regions of Africa and later Southeast Asia, this climate resilient, C4 crop can now also be found in temperate growing regions of Australia, Europe and the Americas^1–3^. The grain is a popular gluten free substitute, the stalks are juiced for syrup in ethanol production and biomass is used as forage^4–6^. Lignocellulosic biomass from bioenergy sorghums are promising feedstocks for production in areas with marginal fertility^7^. These regions are not suitable for other commodity crops like corn and soybeans because they are prone to periods of extreme heat, drought, or sporadic rainfall^8–10^.

**Figure 1:**
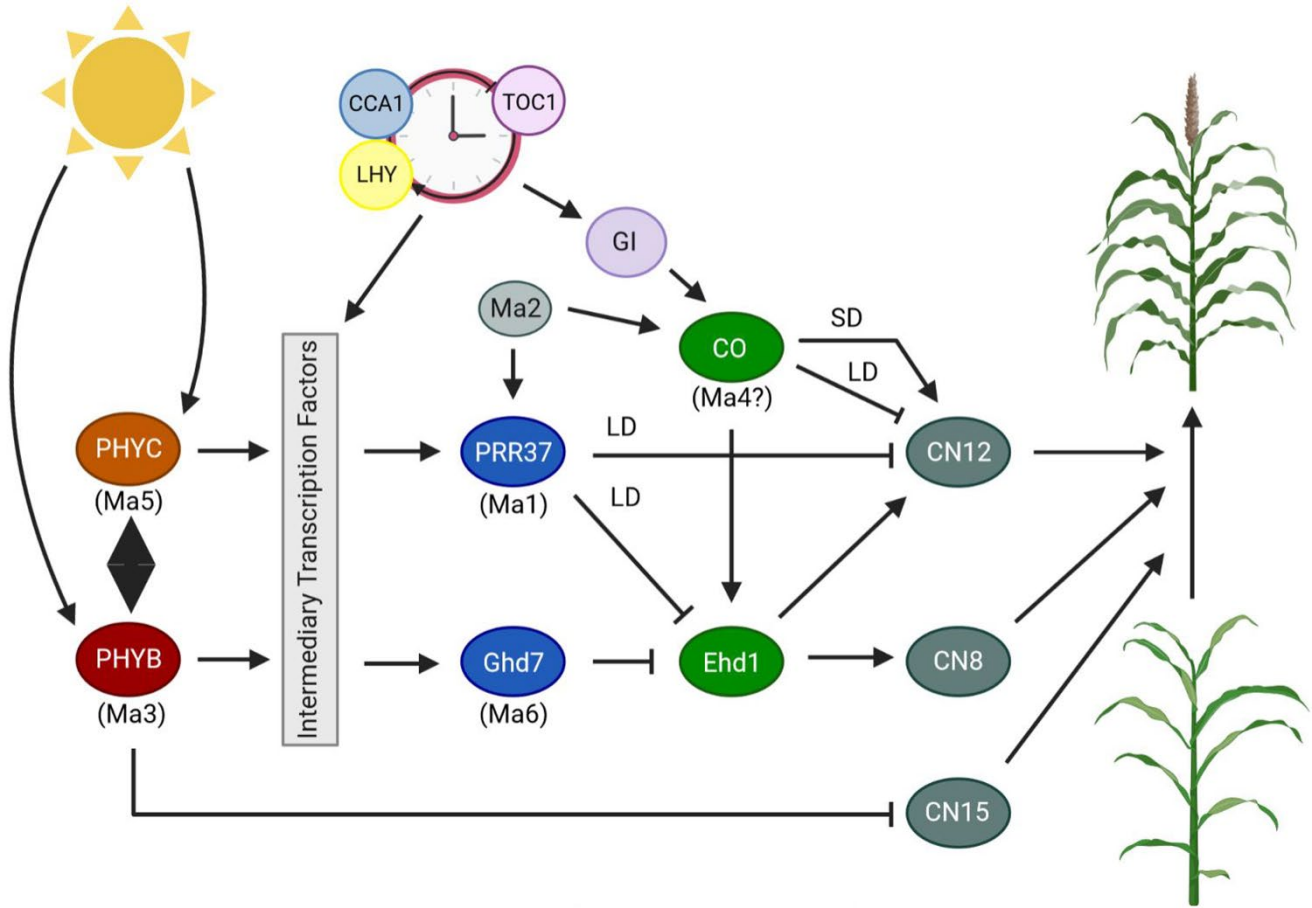
A predicted model of flowering time pathway in sorghum based on GIGANTEA-CONSTANS-FLOWERING LOCUS T (GI-CO-FT) regulatory module, found in Arabidopsis and rice. Light regulated and gated by circadian clock proteins, LATE ELONGATED HYPOCOTYL (LHY), CIRCADIAN CLOCK ASSOCIATED (CCA1) and TIMING OF CAB1 (TOC1). *SbPhyB* may stabilize and interact with *SbPhyC* to inhibit flowering in long days (> 12 h) by activating expression of *SbPRR37* and *SbGhd7*, which results in repression of floral activators Early Heading Date 1 (*SbEHD1*), and FT-like genes *CENTRORADIALIS 8* and *12* (*SbCN8* and *SbCN12*) required for floral initiation. *SbPhyB* facilitates repression of *SbCN15*. Under long days *Ma2* may have an epistatic interaction with *Ma4* to delay flowering by inducing expression of *SbPRR37* and *SbCO* to co-repress the expression of *SbCN12*. Figure adapted and modified from Yang et al.^17^ and Casto et al.^18^. Created using BioRender.com.

In sorghum, inflorescence meristem development is controlled by circadian clock, light quality, phytohormones, developmental stage and temperature^11^. Grain sorghum hybrids flower early (42–90 days after planting) to reduce the risk of exposure to abiotic stress during the reproductive and maturity phases. Sweet sorghums have longer vegetative growth periods (flowering 70–100 days after planting) that allows greater potential for sugar accumulation. Photoperiod sensitive forage and bioenergy sorghums flower extremely late (> 120 days after planting) in temperate environments^12,13^. African sorghum landraces (bicolor, guinea, caudatum, kafir, and durra) were converted from short-day flowering plants (photoperiod of < 11 h) to temperate photoperiod (days with > 12 h daylight), through spontaneous loss of function mutations at six *Maturity* (*Ma1*, *Ma2*, *Ma3*, and *Ma4*^14–16^ and *Ma5* and *Ma6*^12^) loci (Table 1 and Fig. 1). Sorghum accessions with 5 out of 6 functional alleles at the *Ma* loci flower extremely late, approximately 120 days after planting under a temperate photoperiod,^12,13^. Different combinations of alleles at these loci have been used to tune the flowering set period from May 1^st^ to October 1^st^ in temperate zones (latitudes between 31°N and 45°N).

**Table 1:** *Sorghum bicolor Maturity* and *Dwarf* loci information.

Gene ID & Synonyms	Gene Name	Ortholog	GenBank (NCBI)	Location	Coding Strand	Length		Exons
						Transcript	Protein	
Sobic.006G057866 SORBI_3006G057866 Sb06g014570	*Ma1*	*PRR37*	OQU81435	Chr06: 40,304,883–40,316,799	+	4373 bp	739 aa	8
Sobic.002G302700 SORBI_3002G302700	*Ma2*	*–*	KXG36224	Chr02: 67,882,606–67,888,127	+	1677 bp	401 aa	11
Sobic.001G394400 SORBI_3001G394400 Sb01g037340	*Ma3*	*PHYB*	EER94971	Chr01: 68,034,103–68,043,358	−	5508 bp	1178 aa	4
Sobic.001G087100 SORBI_3001G087100 Sb01g007850	*Ma5*	*PHYC*	OQU90974	Chr01: 6,748,036–6,753,421	−	4372 bp	1135 aa	4
Sobic.006G004400 SORBI_3006G004400 Sb06g000570	*Ma6*	*GHD7*	OQU81054	Chr06: 697,459–700,101	+	741 bp	246 aa	3
Sobic.009G229800 SORBI_3009G229800 Sb09g028275	*Dw1*	*–*	KXG22524	Chr09: 57,038,653–57,041,166	+	1533 bp	528 aa	2
Sobic.006G067700 SORBI_3006G067700 Sb06g015430	*Dw2*	*KIPK*	OQU81510	Chr06: 42,803,037–42,807,520	−	4145 bp	809 aa	2
Sobic.007G163800 SORBI_3007G163800 Sb07g023730	*Dw3*	*PGP1* *ABCB1*	OQU80682	Chr07: 59,821,905–59,829,921	−	5304 bp	1541 aa	5

List of known *Ma* and *Dw* loci identification, name, ortholog, GenBank identifier, chromosome location, coding strand, transcript and protein length and number of exons.

**Figure 2:**
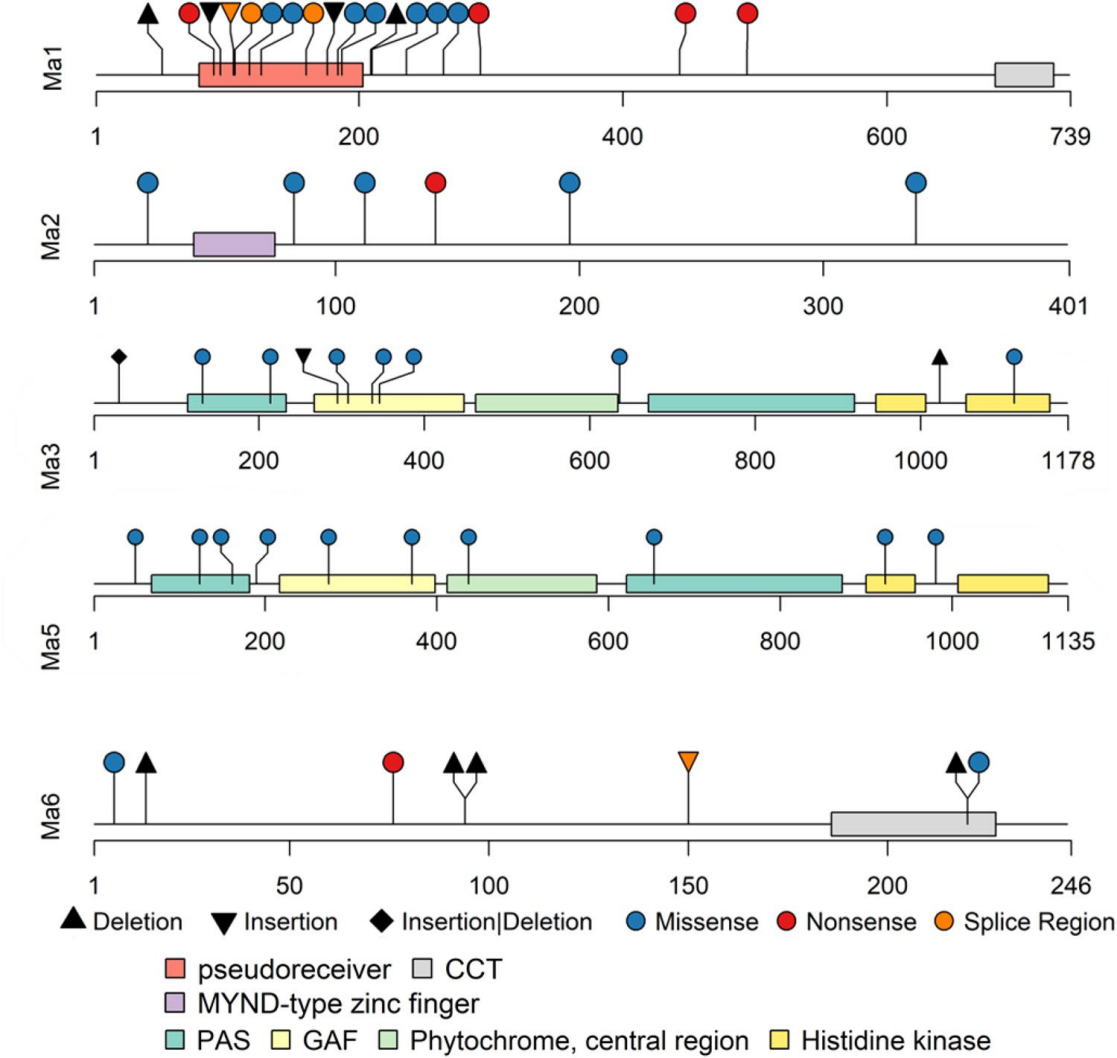
*Sorghum bicolor* lollipop plot schematics for *Maturity* genes exons (*Ma1, Ma2, Ma3, Ma5, Ma6*) with predicted protein motifs^27^ labeled below. Select deleterious genomic variants are flagged at known locations along the length of the peptide (scale below each plot)^28^. Triangles represent insertions (point down) and deletions (point up), insertion or deletion a diamond, circles indicate a point mutation and color corresponds to the predicted peptide changes and effect on protein (blue missense, red nonsense, orange located in splice region).

Most of the *Ma* genes have been shown to encode components that are involved in sensing photoperiod or transmitting a floral repressive signal under nonpermissive conditions, except for *Ma4* that has not been identified, but maps to chromosome 10 (Fig. 1)^18^. *Ma1* is located on chromosome 6 (Sobic.006G057866) and encodes a PSEUDORESPONSE REGULATOR 37 protein, that includes an N-terminal pseudoreceiver (residues 99–207) and C-terminal CCT [CONSTANS (CO), CO-like, and TIMING OF CAB1 (TOC1)] motif (residues 682–727) (Fig. 2). This locus strongly affects flowering time photoperiod sensitivity^19^. *Ma2* is located on chromosome 2 (Sobic.002G302700) and encodes a SET (Suppressor of variegation, Enhancer of Zeste, Trithorax) and MYND (Myeloid-Nervy-DEAF1) motif containing protein, which is a member of SMYD (SET and MYND together) protein family (Fig. 2)^18^. The SET motif can methylate histone lysine residues with roles regulating chromatin state, transcription, signal transduction, and cell cycling^20^. The MYND motif comprises a DNA binding zinc-finger^21^. *Ma3* and *Ma5* encode phytochromes B and C apoproteins and are found on chromosome 1 (Sobic.001G394400 and Sobic.001G087100 respectively) (Fig. 2)^17,22^. These photoreceptors allow plants to detect red and far-red light^23^. The fully assembled holoprotein includes a chromophore covalently attached to the apoprotein. An N-terminal photosensory motif comprised of PAS (PER, ARNT and SIM) and GAF (cGMP phosphodiesterase, adenylate cyclase, Fh1A) to transduce a light signal, along with PHY (phytochrome-specific GAF-related), form a “light-sensing knot” (Fig. 2)^24^. The C-terminal dimerization moiety includes two PAS motifs and HKRD (histidine-kinase-related domain) for dimerization and nuclear localization (Fig. 2)^17^. *Ma6* located on chromosome 6 (Sobic.006G004400) encodes a *Grain number, plant height and heading date 7* (*Ghd7*) ortholog^25^. Like PRR37, GHD7 contains a CCT motif whose protein family members are involved in the transcriptional complex that regulates flowering (Fig. 2)^26^.

Genes from the flowering time pathway are found in most vascular plants, even non-flowering plants; however, their regulation has diverged. *SbPhyB* inhibits flowering in long days by activating expression of *SbPRR37* and *SbGhd7*, resulting in repression of floral activators *Early Heading Date 1* (*SbEHD1*), and *FT*-like genes *CENTRORADIALIS 8* and *CENTRORADIALIS 12* (*SbCN8* and *SbCN12*) required for floral initiation^17,19,29–31^. *PhyB* increases *PhyC* stability and chromophore-containing *PhyB*:*PhyC* heterodimers are required for *PhyC* activity in Arabidopsis and rice. *SbPhyC* is also epistatic to *SbPRR37* and *SbGhd7*^17^. SbPRR37 together with SbGHD7, modulates photoperiod sensitivity and floral repression in an additive mechanism. Under long days, *Ma2* delays flowering by inducing the expression of *SbPRR37* and *CONSTANS* (*SbCO*), which *Ma4* may encode^32,33^, and their gene products co-repress expression of *SbCN12*^17^. Under long days, *SbGhd7* acts as a strong repressor of flowering, increasing photoperiod sensitivity by inhibiting expression of floral activators *SbEHD1*, *SbCN12* and *SbCN8*, hence is light dependent and gated by circadian clock^34,35^. *SbGhd7* is a known component of the photoperiod *GIGANTEA-CONSTANS-FLOWERING LOCUS T* (*GI-CO-FT*) regulatory module for short day rice and long day Arabidopsis^25,36,37^. There is a synergistic effect between constituents of *SbGhd7* and *SbPRR37* loci to enhance photoperiod sensitivity and delay flowering when functional alleles are present at both loci. In contrast, sorghum flowers early when loss of function alleles are present at both loci (Fig. 1)^17^.

**Figure 3:**
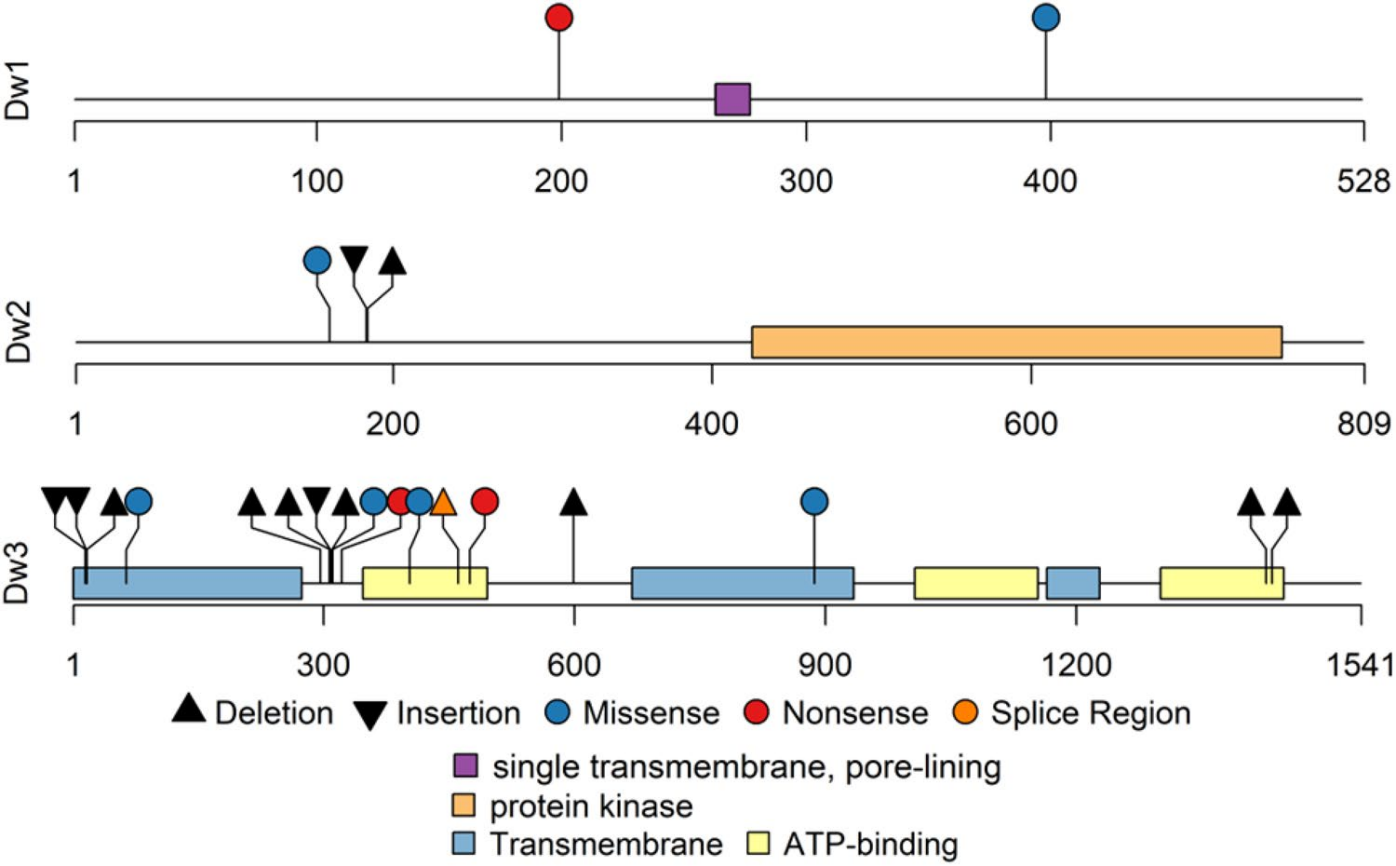
*Sorghum bicolor* lollipop plot schematics for *Dwarf* gene exons (*Dw1, Dw2,* and *Dw3*) with predicted protein motifs^27^ labeled below. Select deleterious genomic variants are flagged at known locations along the length of the peptide (scale below each plot)^28^. Triangles represent insertions (point down) and deletions (point up), circles indicate a point mutation and color corresponds to the predicted peptide changes and effect on protein (blue missense, red nonsense, orange located in splice region).

Flowering time is positively correlated with height in sorghum, thus early flowering plants have short statures, reducing lodging risk and enabling machine harvest. Reduced height is achieved with shorter internode lengths while leaf area and maturity are unchanged^38^. The genes encoded at three of the four *Dwarf* (*Dw1, Dw2,* and *Dw3*) loci^38^ have been identified (Table 1 and Fig. 3). The *Dw4* locus occurs at approximately 6.6 Mb on chromosome 6 but has not been further characterized^39,40^. *Dw1* (Sobic.009G229800), located on chromosome 9, encodes a positive modulator of brassinolide (BR) signaling^41^ by inhibiting nuclear localization of signaling repressor, BRASSINOSTERIOID INSENSITVE 2 (BIN2) that prevents cell proliferation in internodes^42^. The *dw1* mutants have reduced internode cell proliferation activity, a synergistic phenotype with *Dw3,* which can also result in reduced internode length^43^. The *Dw2* locus is located on chromosome 6 (Sobic.006G067700) and encodes a protein kinase with similarity to kinesin-like calmodulin-binding protein (KCBP)-interacting protein kinase (KIPK) and is a member of the AGC protein kinase family in Arabidopsis^44^. *Dw2* phosphorylates proteins involved in lipid signaling, endomembrane trafficking, hormone, light, and receptor signaling, and photosynthesis^45^. *Dw3* is located on chromosome 7 (Sobic.007G163800) and encodes an ATP-binding cassette type B1 auxin efflux transporter (ABCB1)^46^. The height reduction phenotype found in *dw3-ref* mutant is from a loss-of-function P-glycoprotein; that decreases polar auxin transport in seedlings, reduces stalk height (from shortened lower internodes), increased stem thickness, and alters stalk vasculature^47^.

Current and emerging biotechnological advancements offer promising opportunities for improving sorghum bioenergy and green chemical applications. Next-generation sequencing has become an essential tool for obtaining large amounts of genetic information; although, available studies generally discuss the whole genome and not individual loci and phenotypes. Two recent sorghum whole genome sequencing studies were published with a diverse, worldwide collection (n = 499) and sorghum association panel (SAP; n = 400)^48,49^. Both studies used Illumina short read (2 × 100–150 bp) technology: sequencing depth ranged from 0.03 to 116.06$\times$ (average 17.73$\times$) for the Lozano study and 25 to 72$\times$ (average 38$\times$) for the Boatwright study. In this study, genomic variants in the known *Maturity* and *Dwarf* loci were analyzed to identify novel loss of function alleles and characterize the sorghum germplasm using the predicted variants. The sorghum germplasm presented is a valuable breeding resource for manipulating flowering time and plant height to temperate environments and functional ideotypes: grain, forage/biomass, and sweet.

## Results

**Figure 4:**
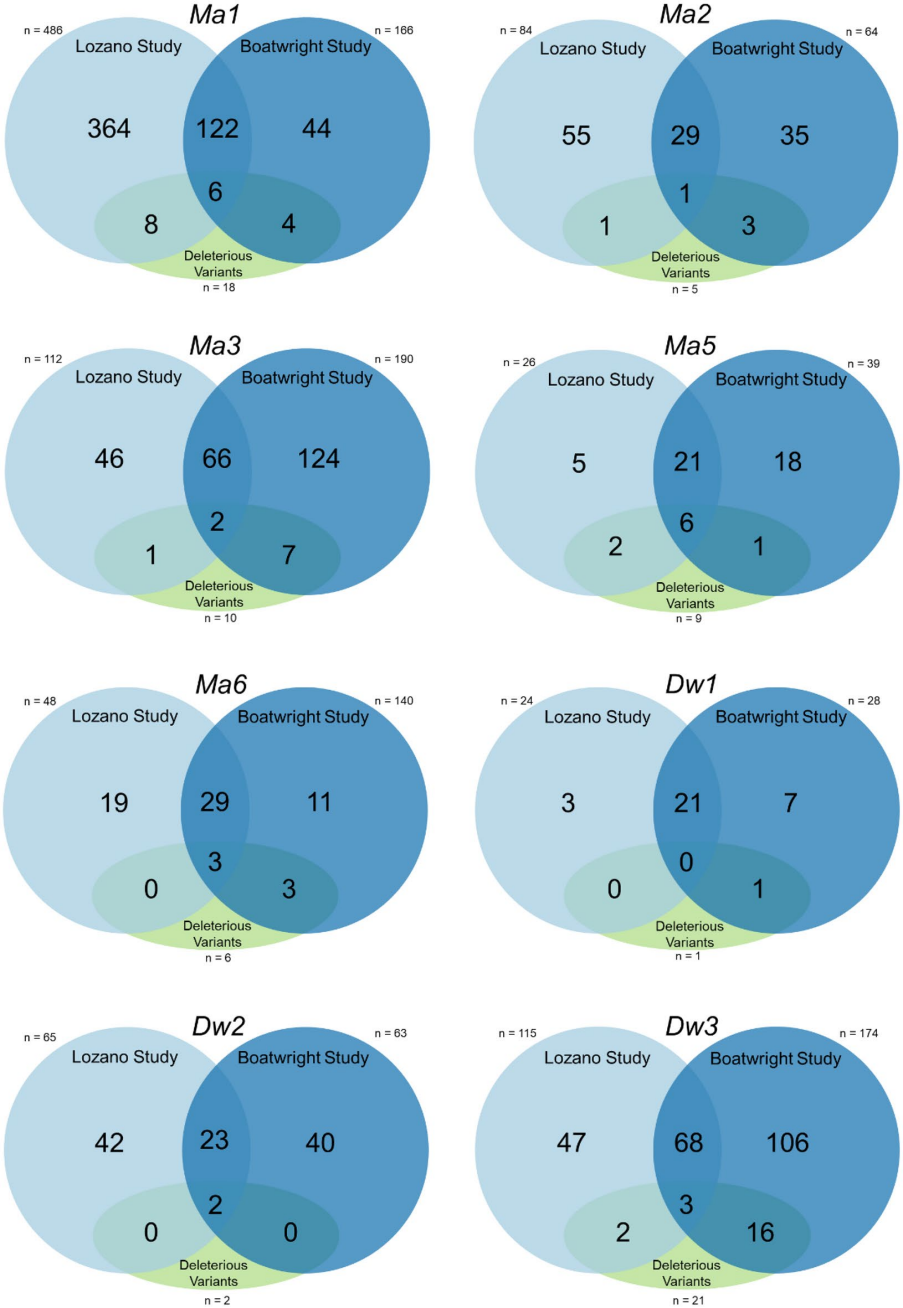
Set diagrams indicating total number of whole-genome sequencing predicted variants from Lozano et al.^48^ (left set) and Boatwright et al.^49^ (right set) for *Ma1*, *Ma2*, *Ma3*, *Ma5*, *Ma6*, *Dw1*, *Dw2*, and *Dw3* loci. The bottom set is a count of deleterious variants and intersecting the unique deleterious variants in each study^50^.

**Figure 5:**
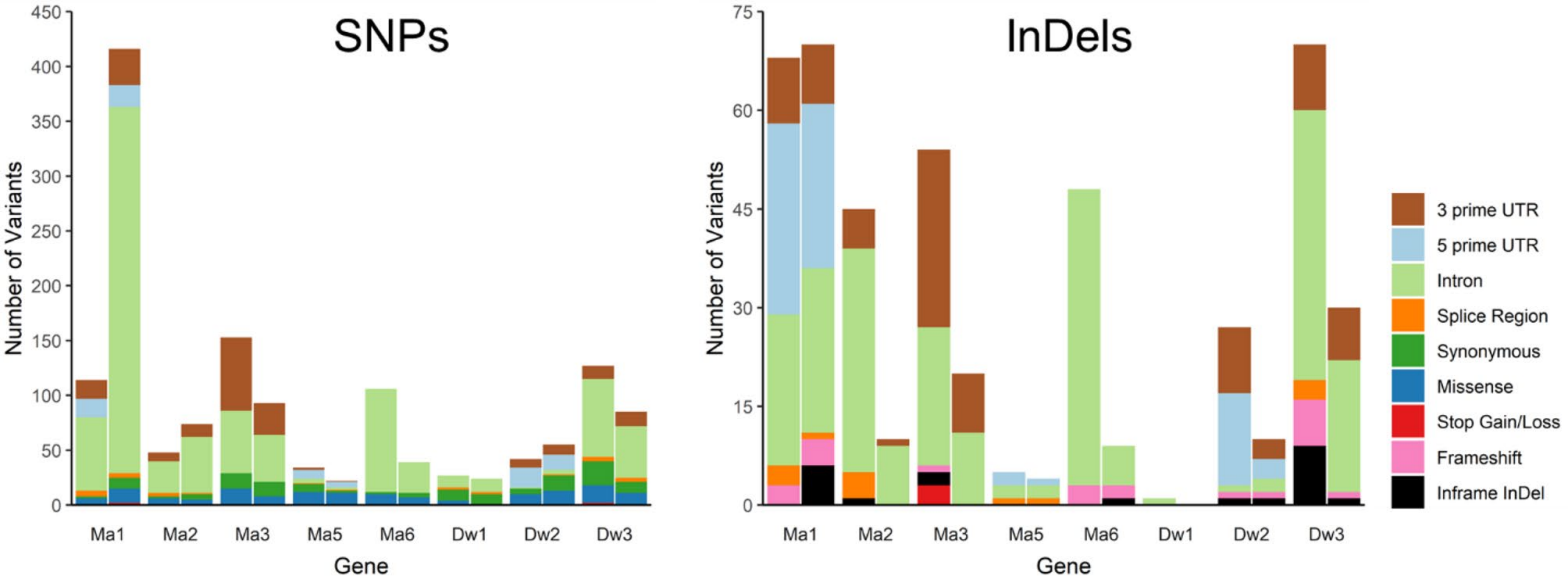
Number of SNPs and InDels for each predicted variant effect. The whole-genome sequencing data from Lozano et al.^48^ (left bar) and Boatwright et al.^49^ (right bar) for *Ma1*, *Ma2*, *Ma3*, *Ma5*, *Ma6*, *Dw1*, *Dw2*, and *Dw3* genes.

To analyze the sorghum loci controlling flowering time and plant height, SNPs and InDels identified in *Maturity* (*Ma1, Ma2, Ma3, Ma5,* and *Ma6*) and *Dwarf* (*Dw1, Dw2,* and *Dw3*) loci from two whole genome sequencing studies^48,49^ were used (see Figs. 4, 5 and Supplementary Tables S1–S9 online). Across all loci 1445 gene variants were identified. In the Lozano dataset^48^, 960 variants were found across these eight genes and 37 were predicted to have a high protein impact or were considered deleterious via SIFT prediction (Figs. 4 and 5)^50^. The low sequencing depth from several lines in this study did not impact the identification of novel alleles. For the Boatwright study^49^, 864 variants were reported; 58 had protein impact or were considered deleterious (Figs. 4 and 5)^50^. The Lozano study had 482 unique sorghum lines, whereas the Boatwright study included the entire 400 sorghum association panel. When compared together an overlap of 22 sorghum lines were found (see Supplementary Table S9 online). This resulted in 860 total sorghum lines for this study.

### Deleterious genomic variants of maturity genes

*Ma1* has seven previously characterized^19,51^ mutant alleles (*Sbprr37-1* or *prr27*^*Milo*^, *Sbprr37-2* or *prr37*^*Kafir-1*^, *Sbprr37-3* or *prr37*^*Kafir-2*^*, prr37*^*Sudangrass*^, *prr37*^*Feterita*^, *prr37*^*Durra*^, and *prr37*^*Broomcorn*^) that can be identified using the resequencing datasets. Between the two studies, 530 unique variants were predicted from a total 652 variants (Fig. 4). Nomenclature from previous studies was used to label the corresponding alleles^19,51^. *Sbprr37-1* or *prr37*^*Milo*^ contains a 1-bp deletion upstream of the pseudo-receiver motif, likely resulting in a null or amorphic allele (Fig. 2). *Sbprr37-2* or *prr37*^*Kafir-1*^ has a missense mutation p.(Lys184Asn) whose substitution of an uncharged asparagine for a positively charged lysine likely alters functionality of the pseudoreceiver motif. *Sbprr37-3* or *prr37*^*Kafir-2*^ contains both a nonsense mutation p.(Gln270Ter) before the CCT motif, and a missense mutation p.(Lys184Asn). The *Sbprr37-3* or *prr37*^*Kafir-2*^ allele is the result of a nonsense mutation at p.(Gln292Ter), and other characterized nonsense variants include *prr37*^*Durra*^ p.(Ser443Ter) and *prr37*^*Broomcorn*^ p.(Gln494Ter). Several other race specific alleles are also characterized^20^: *prr37*^*Sudangrass*^ p.(Ile126Lys), *prr37*^*Feterita*^ p.(Gly177RfsTer4), *prr37*^*Durra*^ p.(Ser^443^Ter), and *prr37*^*Broomcorn*^ p.(Gln494Ter). The *prr37*^*Sudangrass*^ and *prr37*^*Feterita*^ alleles are located in a conserved pseudoreceiver motif. An additional 11 alleles with predicted deleterious mutations (Figs. 4 and 5) include four nonsense, two 1-bp insertions, one 1-bp deletion, two splice site, and eight missense variants (see Supplementary Table S1 online). The nonsense mutation p.(Cys90Ter) in PI 562717 results in a predicted truncated protein, beginning at the pseudoreceiver motif (Fig. 2), which also contains nonsense mutation p.(Ser443Ter). Two 1-bp insertions p.(Val95GlnfsTer16) and p.(Gly177ArgfsTer4) and a 1-bp deletion p.(Gly^209^GlufsTer74) were predicted to result in frameshifts, found in 22 sorghum accessions. Splice acceptor site variants are predicted to affect splicing outside of the consensus sites in the + 3 to + 5 range at the beginning of introns and from -3 to -10 at the end of introns^52^. Two missense splice region variants in *Ma1* were predicted after intron 1 p.(Val106Asp) in lines PI 656035 and PI 656050 and intron 2 p.(Met160Leu) in line PI 35038. Six additional missense amino acid changes were predicted: p.(Asn117Thr), p.(Ile126Lys), p.(Arg187Cys), p.(Ser210Cys), p.(Asp236Tyr), and p.(Trp264Arg). These missense mutations were estimated to have a deleterious effect on protein function due to their homology to conserved residues in orthologous genes and nine variants are in the conserved pseudoreceiver motif. Less than half (355) of the 860 lines sequenced in these studies predicted a fully functional *Ma1* allele, while the *Sbprr37-1* or *prr27*^*Milo*^, *Sbprr37-2* or *prr37*^*Kafir-1*^, and *Sbprr37-3* or *prr37*^*Kafir-2*^ alleles^19,51^ were found in 182, 156 and 45 lines, respectively (see Supplementary Table S1 online). The allelic composition at *Ma1* locus was unable to be determined for 80 lines due to poor or missing data.

There are two characterized alleles of *ma2*. A likely amorphic allele with a nonsense mutation p.(Leu141Ter) in the third exon outside the characterized MYND motif (38 M, 44 M, SM60, 60 M, SM 80, and 80 M)^18^ was found in seven lines. A second characterized missense allele is predicted p.(Met83Thr) to be deleterious by PROVEAN (Protein Variation Effect Analyzer) and is only found in IS3614-2^18^. Of the 119 gene variants identified, 5 were predicted to deleteriously affect Ma2 protein (Figs. 4 and 5). We identified four new variants p.(Gly23Ala), p.(Leu112Ile), p.(Tyr196Phe), and p.(Arg338Trp) in 21 accessions (see Supplementary Table S2 online). None of these missense mutations were predicted to alter amino acid residues within MYND-type zinc-finger motifs (Fig. 2). Most lines (832) in the datasets appeared to have a functional allele at *Ma2* (see Supplementary Table S9 online).

*Ma3* has two characterized loss of function alleles^17^ and only 40 lines were predicted to have *ma3* loss of function alleles (see Supplementary Table S9 online). *SbphyB-1* (previously *Ma3*^*R*^ or *Ma3*^*Ryer*^; 58 M) allele has a 1-bp deletion p.(Asn1023MetfsTer11) that causes a frameshift resulting in a termination codon 30-bp downstream; one line was predicted to have this *Sbphyb-1* allele. The *SbphyB-2* (IS3620C) mutation contains a 3-bp in-frame insertion p.(His31dup) or deletion p.(His31del) and two missense mutations p.(Asp308Gly) and p.(Leu1113Val) (Fig. 2). Eight lines were predicted to have a *Sbphyb-2* allele (see Supplementary Table S9 online). The first substitution p.(Asp308Gly) changes an amino acid in the conserved GAF motif; the residue change has a ‘Sorting Intolerant From Tolerant’ (SIFT) prediction score of 0.1 indicating moderate intolerance^17,50^. There were 236 total gene variants between the two studies for *Ma3* gene, but only 6 mutations were predicted to have deleterious effects (Figs. 4 and 5). Five deleterious variants p.(Asp636Tyr), p.(Pro346Leu), p.(Arg337Gln), p.(Lys214Asn), and p.(Leu132Phe) were found in 13 lines. A large insertion of varying length (89–121 bp) within the GAF motif results in several frameshift variants, p.(Glu295AlafsTer16), p.(Glu295AlafsTer19), or p.(Glu295GlyfsTer25), detected in five heterozygous lines (see Supplementary Table S3 online). Two missense variants p.(Lys214Asn) and p.(Leu132Phe), were predicted to alter amino acids in the PAS fold-2 motif, and two other missense variants, p.(Pro346Leu) and p.(Arg337Gln), were predicted to alter amino acids in the GAF motif (Fig. 2). There were no predicted deleterious *Ma3* alleles in 820 of the 860 accessions analyzed.

The *SbphyC-1* (R.07007) allele is the only *Ma5* mutant characterized^17^. There are four missense mutations: two located in the PAS motif p.(Gly124Val) and p.(Gly162Arg), one in the PAS-GAF loop motif p.(Val190Ala) of exon 1, and one in the HKRD motif p.(Glu922Asp) of exon 2 (Fig. 2). However, the phenotypes of only two of the missense mutations p.(Gly124Val) and p.(Glu922Asp) differ from the wildtype phenotypes of 90 M and 100 M, where both p.(Gly162Arg) and p.(Val190Ala) are present; thus, would likely not confer a change to the flowering time phenotype^17^. There were 44 genomic variants identified in *Ma5*, but only nine of them were predicted to have a deleterious effect on the protein (Figs. 4 and 5). The characterized *Ma5* allele, *SbphyC-1*^17^ was found in six lines (see Supplementary Table S9 online). Six newly identified, missense variants p.(Gly981Asp) [8 lines], p.(Thr653Ser) [PI 656078], p.(Met437Thr) [25 lines], p.(Thr371Ile) [PI 586430], p.(Gln274Glu) [4 lines], and p.(Ser49Tyr) [8 lines], were predicted to be deleterious (see Supplementary Table S4 online). The p.(Met437Thr) SNP is in the GAF motif, and p.(Thr653Ser) is in the PAS fold motif of the protein (Fig. 2).

Of the 159 total gene variants identified in *Ma6*, only six are predicted to have deleterious effects on the protein (Figs. 4 and 5). Amorphic allele *Sbghd7-1* includes a 5-bp insertion (GTCGA) in exon 1 resulting in a frameshift before the CCT motif towards the end of exon 1 p.(Glu94AspfsTer6) (Tx623, 100 M, SM100, BTx406) (Fig. 2)^25^. We identified 385 sorghum lines with the *Sbghd7-1* mutation whereas 281 lines had wildtype alleles at *Ma6*, but 165 lines were unable to be characterized due to incomplete or missing data (see Supplementary Table S9 online). An alternate *Sbghd7-1* allele p.(Glu94SerfsTer77) located at the same position is the result of a 4-bp deletion and was identified in three lines. *Sbghd7-2* is a hypomorphic allele resulting from a large insertion within the second intron (Red Kafir, Hegari, Double Dwarf Feterita, and Rio), whose size and impact have not been precisely determined^25^. Our analyses identified 21 lines with three variable length insertions (18–56 bp, 10 bp and 30–49 bp, respectively) in the second intron region at a nearly identical position (Chr06: 698,157, 698,160, and 698,179) to the ‘Rio’ *Sbghd7-2* allele, where the large insertion was previously described^25^. These small insertions are likely hallmarks of the *Sbghd7-2* allele, but the large insertion could not be fully characterized using short read resequencing mapped to a reference genome (BTx623). Newly identified deleterious variants found include two missense mutations p.(Cys6Tyr) [2 heterozygous lines PI 576347 and PI 576348] and p.(Arg220Gln) [PI 533965], two frameshift deletions p.(Cys14ThrfsTer154) [PI 534046 and PI 576437] and p.(Arg220GlyfsTer2) [3 lines] and a nonsense variant p.(Gln76Ter) [PI 655981] (see Supplementary Table S5 online). The CCT motif of the protein has two mutations predicted to affect the protein (Fig. 2): the 1-bp deletion p.(Arg220GlyfsTer2) and missense p.(Arg220Gln).

### Deleterious genomic variants of dwarf genes

The *Dw1* locus had the fewest number of total gene variants (31) of the accessions analyzed (Figs. 4 and 5). The *dw1* allele contains a nonsense mutation at p.(Lys199Ter)^41^, which is likely an amorphic allele (Fig. 3). However, only one additional predicted deleterious variant p.(Gln398His) was detected. The p.(Gln398His) missense variant was heterozygous in two lines (PI 576375 and PI 609456), but no homozygous alleles were identified (see Supplementary Table S6 online). There are 287 lines with the *dw1* nonsense mutation p.(Lys199Ter) among the two resequencing datasets, 554 lines wildtype for *Dw1*, and 17 lines unable to be characterized due to missing or incomplete data (see Supplementary Table S9 online).

There were 105 total gene variants found in *Dw2*, but only 2 were predicted to have deleterious effects on the gene product (Figs. 4 and 5). The single characterized *dw2* allele p.(Leu184IlefsTer8) contains 2-bp deletion between amino acid position 183 and 184 in exon 1 resulting in a frameshift, hence likely encodes an amorphic allele (Fig. 3)^44^. Almost one third (261) of the lines were identified as having the *dw2* allele while 571 were wildtype and 21 were not characterized due to missing or incomplete data (see Supplementary Table S9 online). A second allele p.(Glu183AspfsTer27) was identified in two lines PI 576396 and PI 656033 at the same location as the *dw2* allele. One newly identified missense variant p.(Ser160Cys) was found in 14 lines (see Supplementary Table S7 online).

There are three previously characterized *dw3* alleles found in exon 5. One (*dw3-ref*) contains an 882 bp duplication event, which reverts at a frequency 0.1–0.5% likely due to unequal crossing-over during DNA replication^46^. The others *dw3-sd1* p.(Leu1435GlyfsTer120) a 2-bp deletion and *dw3-sd2* p.(Gln1174_Arg1175del) a 6-bp deletion located in the C-terminal ABC transporter signature motif (‘LSGGQ’), a highly conserved region with no amino acid sequence variation among eukaryotes^47,53^. There were 221 gene variants identified in the *Dw3* gene, with 21 predicted to have a deleterious effect on the gene product (Figs. 4 and 5). Given the large size of the duplicated repeat (882 bp) in the previously characterized *dw3-ref* allele^46^, we are unable to identify the reported duplication event in any lines from these two datasets (see Supplementary Table S9 online). Two lines (PI 533810 and PI 533927) were heterozygous for the *dw3-sd1* allele p.(Leu1435GlyfsTer120)^46^, but no lines were identified as homozygous for this allele. There were five frameshift variants, a 137 bp deletion p.(Arg311GlnfsTer37) [5 lines], one 2- to 4-bp frameshift deletion p.(Cys17TrpfsTer123) [7 lines] or p.(Cys17AspfsTer54) [9 lines] respectively), a 2-bp deletion p.(Met599AsnfsTer956) [heterozygous PI 576426], and a 1-bp deletion p.(Gly307AlafsTer18) [5 lines]. Three nonsynonymous SNP variants p.(Gly887Arg) [3 lines], p.(Arg403Pro) [6 lines], and p.(Glu64Val [PI 291246 and PI 656047], two nonsense SNPs p.(Gln475Ter) [4 lines] and p.(Glu322Ter) [4 lines] where a stop codon is gained, and one splice donor variant in intron 4 (PI 656078) were predicted to be deleterious across 38 accessions (see Supplementary Table S8 online). Two missense variants p.(Glu64Val) and p.(Gly887Arg) and one 2 p.(Cys17TrpfsTer123) to 4-bp p.(Cys17AspfsTer54) deletion causing a frameshift are in the ABC transporter type 1, transmembrane motif (Fig. 3). The p.(Glu322Ter) and p.(Gln475Ter) nonsense mutations and p.(Arg403Pro) missense mutation are variants predicted to have an impact on the ABC transporter-like, ATP-binding motif. The analyses also confirmed the presence of *dw3-sd2* allele in PI 533957, PI 655978 and PI 655998^53^.

Only 4 accessions among the 860 lines had deleterious mutations at four of the five maturity loci (see Supplementary Table S9 online). PI 533839 has mutant alleles *Sbprr37-1*, *Sbphyb-2*, p.(Gly981Asp) (*ma5*), *Sbghd7-1*, *dw1*, *dw2,* and p.(Cys17TrpfsTer123) (*dw3*) and a wildtype allele at *Ma2*. PI 656021 has mutant alleles *Sbprr37-1*, *ma2*, Sb*phyb-1*, Sb*ghd7-1*, *dw1*, *dw2* and wildtype alleles *SbPHYC-2*. PI 656081 has mutant alleles *Sbprr37-2*, *Sbphyb-2*, p.(Gly981Asp) (*ma5*), *Sbghd7-1*, *dw2*, p.(Cys17TrpfsTer123) (*dw3*) and wildtype alleles *Ma2* and *Dw1*. PI 656116 has mutant alleles *Sbprr37-2*, p.(Arg388Typ) (*ma2*), *Sbphyb-2*, *Sbghd7-1*, *dw2*, p.(Cys17TrpfsTer123) (*dw3*) and wildtype alleles *SbPHYC-1* and *Dw1*. These lines would likely make ideal candidates for development of improved grain sorghum due to the 2 or 3 *dwarf* alleles found in each line and a relatively short time to flowering (60–70 days) under a temperate environment.

### Deleterious alleles and phenotype

To determine the allelic effect on sorghum phenotype, plant height and days to anthesis data was gathered from USDA-ARS Germplasm Resources Information Network (www.ars-grin.gov) and Mural et al.^54^. An Analysis of Variance (ANOVA) test was used to group various allelic means and a t-test to determine the statistical significance (α = 0.05).

Plant height had a significant (p < 0.001) association with the *dwarf* genes and the coefficients used to determine the differences among the alleles (see Supplementary Table S10 online). For *Dw1* the p.(Gln398His) was not found to be significantly different (p = 0.397) compared to reference (*Dw1*) and mutant (*dw1*) phenotype. All the *Dw2* alleles were significant (p < 0.001). The Shapiro–Wilk’s test for normality (W = 0.98786, p = 0.008686)^55^ provides evidence that the data is not normally distributed. A Kruskal–Wallis rank sum test was performed (p < 0.001) (supplementary Fig. S1)^56^.

The number of days to anthesis did not have a considerable association to all *maturity* alleles (see Supplementary Table S11 online). Each individual *maturity* loci had significant alleles (p < 0.001) for days to anthesis. However, when combining all 5 loci, only the *Ma1* alleles were significant (p < 0.001) and some alleles were not viable for statistical inference due to singularities from the dataset. Based on the Shaprio-Wilk’s test (W = 0.99231, p = 0.667)^55^ it can be assumed the population is normally distributed. This result is likely due to the multigenic interaction of six characterized loci controlling the maturity phenotype in sorghum.

## Discussion

The sequence variants characterized in the *Maturity* and *Dwarf* loci are a useful resource for sorghum germplasm development. In these accessions, previously identified alleles along with the genomic variants identified here can be used to manipulate flowering time and plant height. Flowering time is critical for developing plants that flower early to avoid the extreme heat of summer or mature before a killing frost in the fall. Grain sorghum hybrids in the U.S. have a flowering time between 42 and 90 days (photoperiod sensitive) after planting and are relatively short (typically 3-dwarf) for mechanical harvesting and lodging avoidance^38^. Grain hybrids are the result of combining recessive, loss of function alleles at the same *Dw* and *Ma* loci, so the resulting hybrid is homozygous recessive. Some production areas can achieve two or three harvests in a year using short season sorghum^15^. Forage sorghums generally have functional alleles at the *Dw* loci, which maximizes internode length and translates to greater biomass^57^. Forage hybrids may also be photoperiod sensitive, which prevents flowering at temperate latitudes resulting in longer vegetative growth periods and increased biomass production^7^. Lines considered dual purpose produce harvestable grain and stover that may be used in ensiling, and breeders target a longer vegetative growth period with a flowering time towards the end of the growing season to produce both grain and biomass^57^. Each of the sorghum ideotypes (grain, forage/biomass or sweet) were developed for their specific conditions and purposes.

By using available whole genome sequencing and examining these genes, 425 *ma1*, 22 *ma2*, 40 *ma3*, 74 *ma5*, 414 *ma6*, 289 *dw1*, 268 *dw2* and 45 *dw3* alleles were identified, which include ones previously reported. Prior to this work, there have been relatively few alleles characterized at *Ma* and *Dw* loci in sorghum. This study utilized whole genome sequencing datasets of over 800 lines to describe insertion, deletion, missense (nonsynonymous variants), nonsense (stop gain), and splice site mutations that are predicted to impair the function of the encoded protein. Day length has the greatest impact on flowering time in sorghum^18^. *Ma1* had the most deleterious, highly impactful genomic variants compared to the other maturity genes. However, the previously characterized mutant alleles (*Sbprr37-1, Sbprr37-2,* and *Sbprr37-3*) are predicted most frequently in the accessions analyzed. *SbPRR37* is a central component of the flowering regulatory pathway and is influenced by *Ma2* and possibly *Ma4*, downstream of *SbPHYB* (and *SbPHYC*) and co-represses *SbEhd1* with *SbGhd7*^17–19^. Therefore, any substantial change to this protein would strongly affect flowering time. *Ma2* is the least studied of the *Maturity* genes and its role in the flowering time pathway and its interaction with *Ma4* has not been identified^18^. For the *Ma* loci, the fewest genomic variants and predicted deleterious alleles were detected at the *Ma2* locus. This observation may result from the use of the parental line BTx406 (*Sbprr37-1*, *Sbghd7-1*, and recessive for all four *dwarf* loci) in the sorghum conversion program where this line was used as the recurrent parent to convert photoperiod sensitive exotic germplasm into photoperiod insensitive, dwarf lines^58–60^. The *Ma3* locus has several predicted variants; however, many of the lines with *ma3* mutant alleles are the previously characterized *Sbphyb-1* allele. The *Sbphyb-2* allele has three genomic variants corresponding to the reduced day-length sensitivity phenotype in which p.(His31dup) or p.(His31del) and p.(Leu1113Val) are predicated to not impact the protein as severely as p.(Asp308Gly), which alters a conserved residue of the GAF motif, thus likely affecting the function of the protein. Although several genomic variants were identified for the *Ma5* locus that encodes for phytochrome C, very few of these variants were predicted to be loss of function alleles. This result may be because amorphic alleles of *Ma5* are not tolerated in sorghum, and some residual level of function must be maintained in the protein due to the important biological role phytochromes play. The lack of nonsense mutations identified in *Ma5* supports this hypothesis. The *Ma6* locus had the largest InDels (5 and 10 bp) of the *Ma* loci analyzed; a 5-bp deletion was found in the previously characterized *Sbghd7-1* allele^25^. *Ma6* encodes the smallest protein (246 amino acids), and 159 deleterious genomic variants were identified in our analysis, unexpectedly, given the short coding region. Only one new deleterious genomic variant was identified in *Dw1* and *Dw2*, but the previously characterized alleles (*dw1* and *dw2*) were identified in many accessions by our analyses. Many accessions that were sequenced included their common progenitor in their pedigree; for example, Dwarf Yellow Milo (*ma2*, *Sbghd7-1* and *dw1*) is a common progenitor for several lines, which may contribute to the lack of diversity of *ma2*, *Sbghd7-1* and *dw1* mutations among the lines analyzed.

Next-generation resequencing allows researchers to identify single nucleotide variants, insertions/deletions, and copy number changes across many genomes at minimal cost. However, there are limitations to using short sequencing read technologies and assembling these reads to a reference genome to identify variants, for example, the inability to detect repeat regions^60^. Identifying large structural variants substantially greater than the average read length is difficult or not possible without de novo genome assembly. For example, the *dw3-ref* allele contains an 882 bp tandem repeat that was not detectable due to limitations in short read sequencing (100–150 bp) with assembly to a reference genome (Tx623). Several new predicted alleles at *Dw3* were identified in addition to *dw3-ref*, *dw3-sd1* and *dw3-sd2*^46,53^. The *dw3-ref* allele is unstable due to the tandem repeat and reverts at a frequency of 0.1–0.5%^46^; these predicted alleles identified could be used to stabilize this dwarfing phenotype.

Availability of sorghum genomic data is vital for elucidating the genetic architecture of traits and propelling genomics-assisted breeding. Accessions containing previously identified alleles as well as genomic variants not previously-characterized with corresponding germplasm can be used for line development or to study the impact at a locus. This public resource is valuable for fully utilizing the huge variety of sorghum germplasm to develop improved parental lines and hybrids. Developing hybrids with modified flowering and height genes gives breeders flexibility to adapt grain, sweet, and forage/biomass sorghums to specific uses and environments^61^. Having multiple loss of function alleles at critical loci controlling flowering time and plant height will allow for biallelic combinations at each of these loci to maintain heterosis in hybrids.

## Methods

### Whole genome sequencing data analyses

The exon/intron junctions, translation start and stop sites, were obtained from SorghumBase (www.sorghumbase.org) and literature. Additional selected SNP/InDel mutations of all genes were collected from previously published studies^48,49^. In the Lozano study^48^, 499 sorghum lines from a diversity panel were sequenced and about 41 M variants were identified (~ 35 M SNPs and ~ 3.5 M InDels). After quality filtering, ~ 13 M SNPs and ~ 1.8 M InDels were recorded using the reference genome (BTx623)^62^. The Boatwright study^49^ utilized the sorghum association panel (SAP) of 400 accessions and found almost 44 million variants (~ 38 M SNPs and ~ 5 M InDels) but after filtering ~ 19.7 M SNPs and ~ 2.6 M InDels remained. In total, 860 unique accessions were included between both sequencing studies with 22 lines overlapping from both studies. The VCF (Variant Call Format) files were uploaded to the Holland Computing Center at University of Nebraska, Lincoln. UNIX command line operation of intersect was used from the BEDtools utilities suite^63^ to parse the reported variants based on chromosome location.

### Genomic variant analysis

The ‘Sorting Tolerant From Intolerant’ (SIFT) algorithm^50^ predicts whether an amino acid substitution is deleterious to the corresponding protein product for every non-synonymous single nucleotide polymorphism in a coding gene. SIFT uses protein sequence homology to identify conserved amino acids throughout evolution and provides a score of the putative deleterious effect of all possible substitutions at each position in the protein sequence. These scores range from 0 to 1, and positions with a SIFT score < 0.05 are predicted to be deleterious^50^. Variants were separated into four categories: same sense (synonymous) mutations (mutations that do not change the encoded amino acid), tolerated mutations (nonsynonymous missense mutations, SIFT > 0.05), nonsense mutations, and deleterious missense mutations (SIFT < 0.05). Sorghum SIFT annotations were calculated with the EnsemblPlants Variant Effect Predictor Web Tool, a database using the Sorghum_bicolor_NCBIv3 assembly (plants.ensembl.org). Only primary transcripts were considered. Descriptions of sequence variants are based on nomenclature recommendations from the *HGVS nomenclature v*20.05 (varnomen.hgvs.org). This includes p. for prediction of reference amino acid, codon number, alternative amino acid, del for deletion, ins for insertion, fs for frameshift, and Ter for the introduced stop codon and number of codons from the frameshift. Some figures were created using R Statistical Software (v4.2.3)^64^. Lollipop gene schematics were generated using trackViewer R package (v1.34.0)^65^. Bar charts were graphed using the ggplot2 R package (v3.4.2)^66^.

### Statistical analysis of genotype and phenotype

R Statistical Software (v4.2.3)^64^ was used to calculate the linear models for each allele and the corresponding phenotype. The Shaprio-Wilk’s test^55^ for normality was performed and Kruskal–Wallis test^56^ as a non-parametric alternative. Phenotypic data was collected from USDA-ARS Germplasm Resources Information Network (www.ars-grin.gov) and Mural et al.^54^.

### Supplementary Information

Supplementary Information 1.

Supplementary Information 2.

## Data Availability

All data generated or analyzed during this study are included in this published article and its supplementary information files.

## References

[1] Kimber, C. T. in *Sorghum: Origin, History, Technology, and Production* (eds. Smith, C. W. & Frederiksen, R. A.) 3–98 (Wiley, 2000).

[2] Deu M, Rattunde F, Chantereau J (2006). A global view of genetic diversity in cultivated sorghums using a core collection. Genome.

[3] Mullet J (2014). Energy sorghum—A genetic model for the design of C4 grass bioenergy crops. J. Exp. Bot..

[4] Taylor JN, Schober TJ, Bean SR (2006). Novel food and non-food uses for sorghum and millets. Cereal Sci..

[5] Sarath G (2008). Opportunities and roadblocks in utilizing forages and small grains for liquid fuels. J. Ind. Microbiol. Biot..

[6] Wang X (2020). The impacts of flowering time and tillering on grain yield of sorghum hybrids across diverse environments. Agronomy.

[7] Rooney WL, Blumenthal J, Bean B, Mullet JE (2007). Designing sorghum as a dedicated bioenergy feedstock. Biofuel Bioprod. Bior..

[8] Howell TA, Steiner JL, Schneider AD, Evett SR, Tolk JA (1997). Seasonal and maximum daily evapotranspiration of irrigated winter wheat, sorghum, and corn—Southern High Plains. T. ASAE..

[9] Farré I, Faci JM (2006). Comparative response of maize (Zea mays L.) and sorghum (Sorghum bicolor L. Moench) to deficit irrigation in a Mediterranean environment. Agric. Water Manag..

[10] Anderson WF (2016). Dedicated herbaceous biomass feedstock genetics and development. BioEnerg. Res..

[11] Nuñez FD, Yamada T (2017). Molecular regulation of flowering time in grasses. Agronomy.

[12] Rooney WL, Aydin S (1999). Genetic control of a photoperiod-sensitive response in Sorghum bicolor (L.) Moench. Crop Sci..

[13] Packer DJ, Rooney WL (2014). High-parent heterosis for biomass yield in photoperiod-sensitive sorghum hybrids. Field Crop Res..

[14] Quinby JR (1966). Fourth Maturity Gene Locus in Sorghum. Crop Sci..

[15] Quinby, J. R. in *Advances in Agronomy* (ed. Brady, N. C.) **25**, 125–162 (Academic Press, 1974). 10.1016/S0065-2113(08)60780-4

[16] Quinby JR (1975). The genetics of sorghum improvement. J. Hered..

[17] Yang, S. *et al*. Sorghum Phytochrome B Inhibits Flowering in Long Days by Activating Expression of SbPRR37 and SbGHD7, Repressors of SbEHD1, SbCN8 and SbCN12. *PloS One*. **9**(8), e105352 (2014). 10.1371/journal.pone.0105352 10.1371/journal.pone.0105352 PMC4133345 25122453

[18] Casto, A. L. *et al*. Maturity2, a novel regulator of flowering time in Sorghum bicolor, increases expression of SbPRR37 and SbCO in long days delaying flowering. *PloS One*. **14**(4), e0212154 (2019). 10.1371/journal.pone.0212154 10.1371/journal.pone.0212154 PMC6457528 30969968

[19] Murphy RL (2011). Coincident light and clock regulation of pseudoresponse regulator protein 37 (PRR37) controls photoperiodic flowering in sorghum. Proc. Natl. Acad. Sci..

[20] Min J, Zhang X, Cheng X, Grewal SIS, Xu RM (2002). Structure of the SET domain histone lysine methyltransferase Clr4. Nat. Struct. Biol..

[21] Spellmon N, Holcomb J, Trescott L, Sirinupong N, Yang Z (2015). Structure and function of SET and MYND domain-containing proteins. Int. J. Mol. Sci..

[22] Childs KL (1997). The sorghum photoperiod sensitivity gene, Ma3, encodes a phytochrome B. Plant Physiol..

[23] Kami C, Lorrain S, Hornitschek P, Fankhauser C (2010). Light-regulated plant growth and development. Curr. Top. Dev. Bio..

[24] Nagatani A (2010). Phytochrome: Structural basis for its functions. Curr. Opin. Plant Biol..

[25] Murphy, R. L. *et al*. Ghd7 (Ma6) represses sorghum flowering in long days: Ghd7 alleles enhance biomass accumulation and grain production. *Plant Genome*. **7**(2), plantgenome2013–11 (2014). 10.3835/plantgenome2013.11.0040

[26] Li Y, Xu M (2017). CCT family genes in cereal crops: A current overview. Crop J..

[27] Mistry J (2021). Pfam: The protein families database in 2021. Nucl. Acids Res..

[28] Kumar P, Henikoff S, Ng PC (2009). Predicting the effects of coding non-synonymous variants on protein function using the SIFT algorithm. Nat. Protoc..

[29] Cho L-H, Yoon J, Pasriga R, An G (2006). Homodimerization of Ehd1 is required to induce flowering in rice. Plant Physiol..

[30] Tsuji H, Taoka KI, Shimamoto K (2013). Florigen in rice: Complex gene network for florigen transcription, florigen activation complex, and multiple functions. Curr. Opin. Plant Biol..

[31] Wolabu TW (2016). Three FLOWERING LOCUS T-like genes function as potential florigens and mediate photoperiod response in sorghum. New Phytol..

[32] Yang S, Weers BD, Morishige DT, Mullet JE (2014). CONSTANS is a photoperiod regulated activator of flowering in sorghum. BMC Plant Biol..

[33] Kawahigashi H, Yonemaru JI, Kiyosawa A, Mizuno H, Kasuga S (2022). DNA marker analysis of flowering time, semi-dwarf, fertility restorer, and brown midrib genes in sorghum (Sorghum bicolor [L.] Moench). Grassl. Sci..

[34] Schaffer R (1998). The late elongated hypocotyl mutation of Arabidopsis disrupts circadian rhythms and the photoperiodic control of flowering. Cell.

[35] Millar AJ, Carre IA, Strayer CA, Chua NH, Kay SA (1995). Circadian clock mutants in Arabidopsis identified by luciferase imaging. Science.

[36] Park DH (1999). Control of circadian rhythms and photoperiodic flowering by the Arabidopsis GIGANTEA gene. Science.

[37] Xue W (2008). Natural variation in Ghd7 is an important regulator of heading date and yield potential in rice. Nat. Genet..

[38] Quinby JR, Karper RE (1954). Inheritance of height in sorghum. Agron. J..

[39] Miao C, Xu Y, Liu S, Schnable PS, Schnable JC (2020). Increased power and accuracy of causal locus identification in time series genome-wide association in sorghum. Plant Physiol..

[40] Morris GP (2013). Dissecting genome-wide association signals for loss-of-function phenotypes in sorghum flavonoid pigmentation traits. G3-Genes Genom. Genet..

[41] Hilley, J., Truong, S., Olson, S., Morishige, D. & Mullet, J. Identification of Dw1, a regulator of sorghum stem internode length. *PloS One*. **11**(3), e0151271 (2016). 10.1371/journal.pone.0151271 10.1371/journal.pone.0151271 PMC4786228 26963094

[42] Hirano K (2017). Sorghum DW1 positively regulates brassinosteroid signaling by inhibiting the nuclear localization of BRASSINOSTEROID INSENSITIVE 2. Sci. Rep..

[43] Brown PJ, Rooney WL, Franks C, Kresovich S (2008). Efficient mapping of plant height quantitative trait loci in a sorghum association population with introgressed dwarfing genes. Genetics.

[44] Hilley JL (2017). Sorghum Dw2 encodes a protein kinase regulator of stem internode length. Sci. Rep..

[45] Oliver J (2021). The AGCVIII kinase Dw2 modulates cell proliferation, endomembrane trafficking, and MLG/xylan cell wall localization in elongating stem internodes of Sorghum bicolor. Plant J..

[46] Multani DS (2003). Loss of an MDR transporter in compact stalks of maize br2 and sorghum Dw3 mutants. Science.

[47] Li X, Li X, Fridman E, Tesso TT, Yu J (2015). Dissecting repulsion linkage in the dwarfing gene Dw3 region for sorghum plant height provides insights into heterosis. P. Natl. Acad. Sci..

[48] Lozano R (2021). Comparative evolutionary genetics of deleterious load in sorghum and maize. Nat. Plants..

[49] Boatwright JL (2022). Sorghum Association Panel whole-genome sequencing establishes cornerstone resource for dissecting genomic diversity. Plant J..

[50] Ng PC, Henikoff S (2003). SIFT: predicting amino acid changes that affect protein function. Nucl. Acids Res..

[51] Klein RR (2015). Allelic variants in the PRR37 gene and the human-mediated dispersal and diversification of sorghum. Theor. Appl. Genet..

[52] Karbassi I (2016). A Standardized DNA Variant Scoring System for Pathogenicity Assessments in Mendelian Disorders. Hum. Mutat..

[53] Barrero Farfan ID, Bergsma BR, Johal G, Tuinstra MR (2012). A stable dw3 allele in sorghum and a molecular marker to facilitate selection. Crop Sci..

[54] Mural RV (2021). Meta-analysis identifies pleiotropic loci controlling phenotypic trade-offs in sorghum. Genetics.

[55] Shapiro SS, Wilk MB (1965). An analysis of variance test for normality (complete samples). Biometrika.

[56] Kruskal WH, Wallis WA (1952). Use of ranks in one-criterion variance analysis. J. Am. Stat. Assoc..

[57] Pedersen, F. P. & Fritz, J. O. in *Sorghum: Origin, History, Technology, and Production* (eds. Smith, C. W. & Frederiksen, R. A.) 797–810 (Wiley, 2000)

[58] Stephens JC, Miller FR, Rosenow DT (1967). Conversion of alien sorghums to early combine genotypes. Crop Sci..

[59] Klein RR (2008). The effect of tropical sorghum conversion and inbred development on genome diversity as revealed by high-resolution genotyping. Crop Sci..

[60] Bahlo M (2018). Recent advances in the detection of repeat expansions with short-read next-generation sequencing. F1000Research..

[61] Li X, Guo T, Mu Q, Li X, Yu J (2018). Genomic and environmental determinants and their interplay underlying phenotypic plasticity. Proc. Natl. Acad. Sci..

[62] McCormick RF (2018). The Sorghum bicolor reference genome: improved assembly, gene annotations, a transcriptome atlas, and signatures of genome organization. Plant J..

[63] Quinlan AR, Hall IM (2010). BEDTools: A flexible suite of utilities for comparing genomic features. Bioinformatics.

[64] R Core Team. The R project for statistical computing. R: A Language and Environment for Statistical Computing (2023). https://www.R-project.org/.

[65] Ou J, Zhu L (2019). trackViewer: A Bioconductor package for interactive and integrative visualization of multi-omics data. Nat. Methods..

[66] Wickham, H. *ggplot2: Elegant Graphics for Data Analysis*. (Springer, 2016). 10.1007/978-3-319-24277-4

