# Author Correction: Deleterious mutations predicted in the sorghum (*Sorghum bicolor*) *Maturity* (*Ma*) and *Dwarf* (*Dw*) genes from whole‑genome resequencing

**DOI:** 10.1038/s41598-023-45098-z

**Published:** 2023-10-18

**Authors:** Nathan P. Grant, John J. Toy, Deanna L. Funnell‑Harris, Scott E. Sattler

**Affiliations:** 1 Wheat, Sorghum and Forage Research Unit, Agricultural Research Service, United States Department of Agriculture, Lincoln, NE USA; 2 Department of Agronomy and Horticulture, University of Nebraska-Lincoln, Lincoln, NE USA; 3 Department of Plant Pathology, University of Nebraska-Lincoln, Lincoln, NE USA

Correction to: *Scientific Reports* https://doi.org/10.1038/s41598-023-42306-8, published online 03 October 2023

The original version of this Article contained errors.

In the Introduction section, where

“African sorghum landraces (bicolor, guinea, caudatum, kafir, and durra) were converted from short-day flowering plants (photoperiod of > 11 h)”

now reads:

“African sorghum landraces (bicolor, guinea, caudatum, kafir, and durra) were converted from short-day flowering plants (photoperiod of < 11 h)”

Furthermore, in the Results section, where

“There are two characterized alleles of *ma2*. A likely amorphic allele with a nonsense mutation p.(Leu141Ter) in the third exon outside the characterized MYND motif (80 M, 60 M, SM80, SM60, 44 M, 38)”

now reads,

“There are two characterized alleles of *ma2*. A likely amorphic allele with a nonsense mutation p.(Leu141Ter) in the third exon outside the characterized MYND motif (38 M, 44 M, SM60, 60 M, SM 80, and 80 M)”

and where

“Newly identified deleterious variants found include two missense mutations p.(Cys6Tyr) [2 heterozygous lines PI 576347 and PI 576,348] and p.(Arg220Gln) [PI 533,965], two frameshift deletions p.(Cys14ThrfsTer154) [PI 534,046 and PI 576,437]”

now reads:

“Newly identified deleterious variants found include two missense mutations p.(Cys6Tyr) [2 heterozygous lines PI 576347 and PI 576348] and p.(Arg220Gln) [PI 533965], two frameshift deletions p.(Cys14ThrfsTer154) [PI 534046 and PI 576437]”

In addition, under the subheading Deleterious alleles and phenotype, where

“The p.(Gln398His) missense variant was heterozygous in two lines (PI576,375 and PI 609,456), but no homozygous alleles were identified”

now reads,

“The p.(Gln398His) missense variant was heterozygous in two lines (PI 576375 and PI 609456), but no homozygous alleles were identified”

Secondly, where

“A second allele p.(Glu183AspfsTer27) was identified in two lines PI 576,396 and PI 656,033 at the same location as the *dw2* allele.”

now reads,

“A second allele p.(Glu183AspfsTer27) was identified in two lines PI 576396 and PI 656033 at the same location as the *dw2* allele.”

and where

“To determine the allelic effect on sorghum phenotype, plant height and days to anthesis data was gathered from USDA-ARS Germplasm Resources Information Network (www.ars-grin.gov) and Ravi et al.”

now reads,

“To determine the allelic effect on sorghum phenotype, plant height and days to anthesis data was gathered from USDA-ARS Germplasm Resources Information Network (www.ars-grin.gov) and Mural et al.”

Moreover, under the subheading Deleterious genomic variants of dwarf genes, where

“All the *Dw2* alleles were significant (p < 0.001). The Shapiro–Wilk’s test for normality (W = 0.98786, p = 0.008686,55]”

now reads,

“All the *Dw2* alleles were significant (p < 0.001). The Shapiro–Wilk’s test for normality (W = 0.98786, p = 0.008686)[55]”

and where

“Based on the Shaprio-Wilk’s test (W = 0.99231, p = 0.667,55] it can be assumed the population is normally distributed.”

now reads,

“Based on the Shaprio-Wilk’s test (W = 0.99231, p = 0.667)[55] it can be assumed the population is normally distributed.”

Additionally, in the caption of Figure 1, where

“A predicted model of flowering time pathway in sorghum based on GIGANTEA-CONSTANSFLOWERING LOCUS T (GI-CO-FT) regulatory module, found in Arabidopsis and rice.”

now reads,

“A predicted model of flowering time pathway in sorghum based on GIGANTEA-CONSTANS-FLOWERING LOCUS T (GI-CO-FT) regulatory module, found in Arabidopsis and rice.”

Finally, in the Methods section, where

“Some figures were created using R Statistical Software (v4.2.3,64]. Lollipop gene schematics were generated using trackViewer R package (v1.34.0,65]. Bar charts were graphed using ggplot2 R package (v3.4.2,66].”

now reads,

“Some figures were created using R Statistical Software (v4.2.3)[64]. Lollipop gene schematics were generated using trackViewer R package (v1.34.0)[65]. Bar charts were graphed using the ggplot2 R package (v3.4.2)[66].”

and where

“R Statistical Software (v4.2.3,64] was used calculate the linear models for each allele and the corresponding phenotype.”

now reads,

“R Statistical Software (v4.2.3)[64] was used to calculate the linear models for each allele and the corresponding phenotype.”

and where

“Phenotypic data was collected from USDA-ARS Germplasm Resources Information Network (www.ars-grin.gov) and Ravi et al.”

now reads,

“Phenotypic data was collected from USDA-ARS Germplasm Resources Information Network (www.ars-grin.gov) and Mural et al.”

The original Article has been corrected.

