# Supplementary material for: Deleterious mutations predicted in the sorghum (Sorghum bicolor) Maturity (Ma) and Dwarf (Dw) genes from whole-genome resequencing
Source: Sci Rep. 2023 Oct 3;13:16638. doi: 10.1038/s41598-023-42306-8 (PMC10547693; doi:10.1038/s41598-023-42306-8)
Supplement: Supplementary file 2 — Supplementary Information 2. [file 41598_2023_42306_MOESM2_ESM.pdf]

SUPPLEMENTARY INFORMATION

Additional File 1.xlsx

Supplementary Table S1. Identified deleterious mutations of Ma1. Supplementary Table S2. Identified deleterious mutations of Ma2. Supplementary Table S3. Identified deleterious mutations of Ma3. Supplementary Table S4. Identified deleterious mutations of Ma5. Supplementary Table S5. Identified deleterious mutations of Ma6. Supplementary Table S6. Identified deleterious mutations of Dw1. Supplementary Table S7. Identified deleterious mutations of Dw2. Supplementary Table S8. Identified deleterious mutations of Dw3. Supplementary Table S9. List of all sorghum lines with the corresponding genotype for each locus. Supplementary Table S1 – S8 describe the identified deleterious mutation predictions for Ma1, Ma2, Ma3, Ma5, Ma6, Dw1, Dw2, and Dw3, respectively. Labeled with location, variant type, SIFT score or impact on protein function. Some corresponding genotypes are listed for each variant from literature. Supplementary Table S9 list of all sorghum lines with the corresponding metadata and genotype for each locus [1, 2, 3, 4, 5, 6]. § denotes heterozygous for an allele. Supplementary Table S10 Summary ANOVA’s coefficients for plant height and Dwarf alleles. Supplementary Table S11 Summary ANOVA’s coefficients for plant height and Maturity alleles.

Supplementary Fig. S1 A Kuskal-Wallis multiple comparison for the number of mutant *dwarf* loci (none, dw1/dw2 or both) and plant height with p-values adjusted with the Holm method [7]. Figure made using ggstatsplot package [8] in R Statistical Software (v4.2.3; 9)

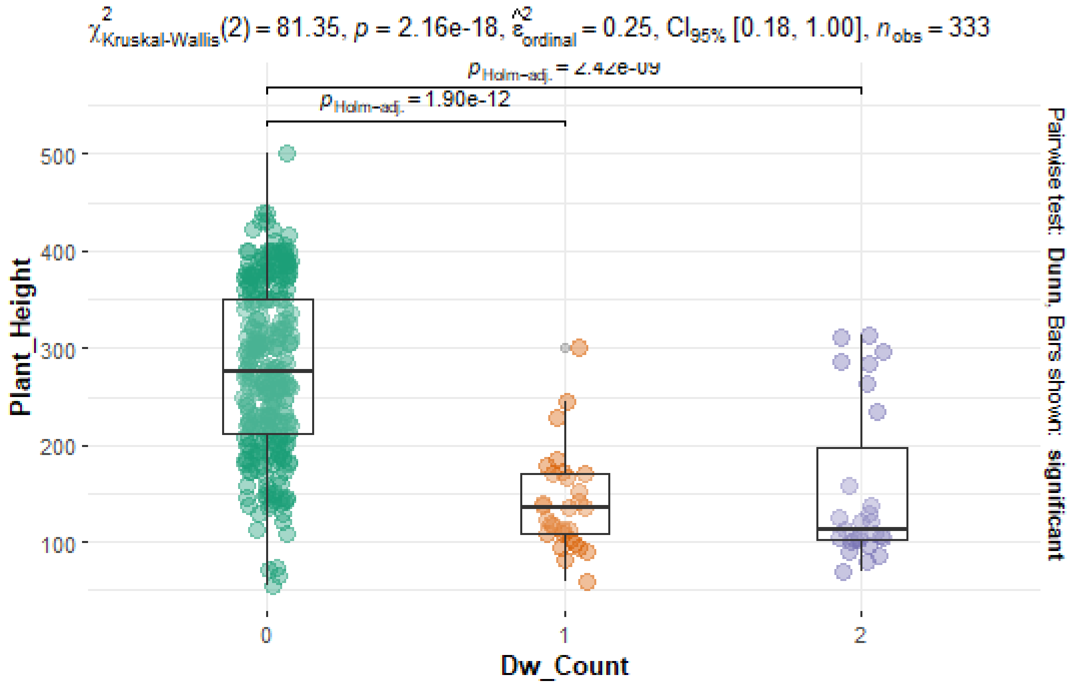

REFERENCES

1. Yang, S. *et al*. Sorghum Phytochrome B Inhibits Flowering in Long Days by Activating Expression of SbPRR37 and SbGHD7, Repressors of SbEHD1, SbCN8 and SbCN12. *PloS One*. **9**(8), e105352 (2014). Doi:10.1371/journal.pone.0105352

2. Casto, A. L. *et al*. Maturity2, a novel regulator of flowering time in Sorghum bicolor, increases expression of SbPRR37 and SbCO in long days delaying flowering. *PloS One*. **14**(4), e0212154 (2019). Doi:10.1371/journal.pone.0212154

3. Murphy, R. L. *et al*. Coincident light and clock regulation of pseudoresponse regulator protein 37 (PRR37) controls photoperiodic flowering in sorghum. *P. Natl. Acad. Sci*. **108**(39), 16469–16474 (2011). Doi:10.1073/pnas.1106212108

4. Murphy, R. L. *et al*. Ghd7 (Ma6) represses sorghum flowering in long days: Ghd7 alleles enhance biomass accumulation and grain production. *Plant Genome*. **7**(2), plantgenome2013-11 (2014). Doi:10.3835/plantgenome2013.11.0040
5. Lozano, R. et al. Comparative evolutionary genetics of deleterious load in sorghum and maize. *Nat. Plants*. **7**(1), 17-24 (2021). Doi:10.1038/s41477-020-00834-5
6. Boatwright, J. L. et al. Sorghum Association Panel whole-genome sequencing establishes cornerstone resource for dissecting genomic diversity. *Plant J*. **111**(3), 888-904 (2022). Doi:10.1111/tpj.15853
7. Dunn, O. J. Multiple Comparisons Using Rank Sums. *Technometrics* **6**(3), 241-252 (1964). Doi:10.1080/00401706.1964.10490181
8. Patil, I. Visualizations with statistical details: The 'ggstatsplot' approach. *J. Open Source Softw*, **6**(61), 3167 (2021). Doi:10.21105/joss.03167
9. R Core Team. The R project for statistical computing. R: A Language and Environment for Statistical Computing (2023). Available at: https://www.R-project.org/.
